# Challenges related to the cases of lepromatous leprosy: a report of two cases

**DOI:** 10.11604/pamj.2022.41.35.32318

**Published:** 2022-01-13

**Authors:** Mohammed Alfaragi, Faisal Ahmed, Waiel Osman, Islam Mustafa, Intisar Almustafi, Rehab Dawoud, Fahad Alrashdi

**Affiliations:** 1 Skin Soft Clinic, Department of Dermatology, Unaizah, Kingdom of Saudi Arabia,; 2 Urology Research Center, Al-Thora General Hospital, Department of Urology, School of Medicine, Ibb University of Medical Science, Ibb, Yemen,; 3 Department of Dermatology, King Saud Hospital, Qassim, Kingdom of Saudi Arabia

**Keywords:** Leprosy, diagnostic errors, Saudi Arabia, case report

## Abstract

Leprosy is a chronic granulomatous disease caused by Mycobacterium leprae with various clinical symptoms. Even though the incidence of leprosy has diminished in Saudi Arabia, it still has not been eliminated. We present two cases of leprosy from India residing in Saudi Arabia that were successfully managed with multidrug therapy. The first patient was a 33-year-old man with a history of enlarging erythematous patch and nose deformity on his face and upper and lower extremities started two years ago. The second patient was a 25-year-old man who presented with multiple hyperpigmented plaques on the upper and lower extremities in the last two years. The patient was firstly misdiagnosed and treated as tinea corporis without improvement. In both patients, the histological study from cutaneous lesions confirmed lepromatous leprosy. This report aimed to introduce the manifestations of leprosy, emphasizing the nose's involvement in the first case and the misdiagnosis of leprosy in the second case.

## Introduction

Mycobacterium leprae is an intracellular pathogen causing leprosy involving the skin and peripheral nerve. Clinical features influence the multitude of lesions and their trends of dispersion. The various clinical forms of leprosy are primarily related to the infection's immunological responses [1]. Leprosy is a treatable illness if diagnosed earlier. Rapid recognition is essential for disability avoidance. The World Health Organization has officially classified leprosy as a forgotten tropical disease, with recent worldwide data showing about 208,619 newly diagnosed leprosy cases [1,2]. Additionally, it was reported that 242 freshly diagnosed leprosy cases were in Saudi Arabia from 2003 to 2012 [3]. Leprosy occurrence in Saudi Arabia is rare. As a result, we present two cases of leprosy that manifested the lepromatous clinical form, highlighting the involvement of the nose in the first case and the misdiagnosis of leprosy in the second case.

## Patient and observation

### Clinical case 1

**Patient information:** a 33 years ancient man from India residing in Saudi Arabia. In March 2017, he presented with enlarging erythematous patch and diffuse skin swelling on the face, lower limbs, and trunk, which started two years ago. He also suffered from nasal stuffiness and intermittent nasal bloody discharge. He was otherwise well. The patient did not have any previous history of contact with leprosy patients.

**Figure 1:**
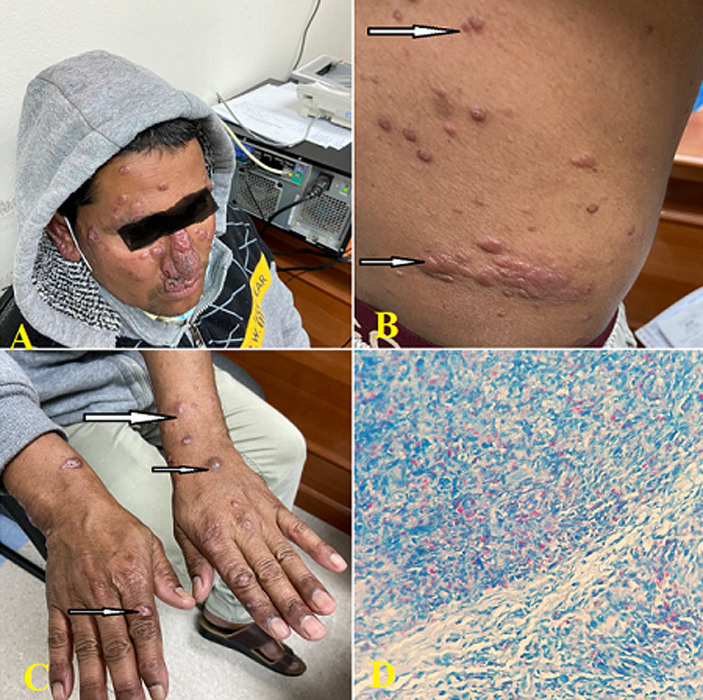
the photos of first case showing; A) multiple nodules on the face and saddle nose; B) the hypochromic areas and multiple nodules on the abdomen (arrow); C) multiple nodules on both hands; D) histopathology showing lepromatous leprosy

**Clinical findings:** physical examination revealed erythematous nodules all over the face, upper and lower extremities, and the back. He also suffers from deformity of the nose (saddle nose). Bilateral thickening of the ulnar nerve associated with loss of sensation above it was noted. All other superficial nerves were normal. The clinical assessment showed no motor changes (Figure 1A, Figure 1B, Figure 1C).

**Diagnostic assessment:** bacilloscopy was done in slite skin smears and histological sections of the cutaneous hypochromic lesion to lead the investigation. The skin smear (nose and reticulated lesion on the thigh) was positive, with a histological study demonstrating a granulomatous inflammatory infiltrate with so many bacilli. These findings pointed to lepromatous leprosy (Figure 1D).

**Treatment:** the patient was treated with Rifampicin, Clofazimine, and Dapsone, followed by 1 mg/kg/day prednisone for neuritis, and continued to improve his condition.

**Follow-up and outcome:** after these manifestations improved, the patient returned to India. He was provided a confirmation letter of his diagnosis and strongly recommended seeking medical treatment and continuing treatment if needed.

### Clinical case 2

**Patient information:** a 25 years old man from India living in Saudi Arabia presented in January 2014 with multiple hyperpigmented and hypopigmented plaques with slightly elevated borders. The patient did not have any previous history of contact with leprosy patients. Firstly, the patient was treated for *Tinea corporis* without improvement, and the final diagnosis was made after the histopathology report.

**Figure 2:**
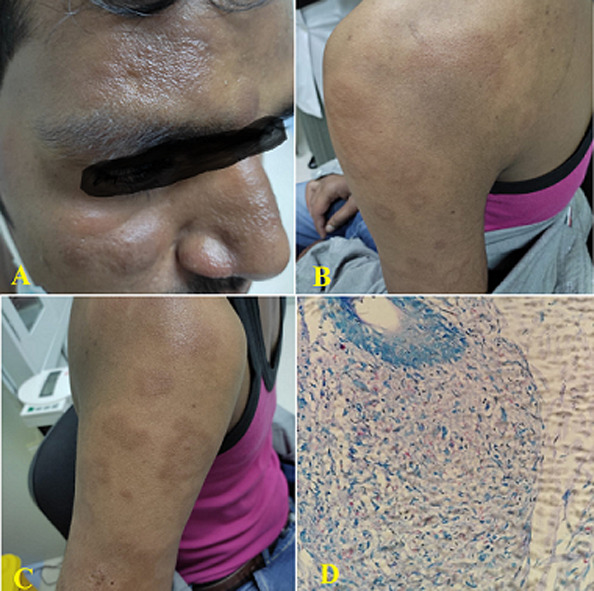
the photos of second case showing; A) the multiple nodules on the face; B) the multiple, soft, and skin-colored superficial nodules; C) the erythematous nodules and hyperpigmented patches; D) histopathology showing lepromatous leprosy

**Clinical findings:** physical examinations demonstrated non-pitting edema of the ears, erythematous nodules, and darkish, hyperpigmented patches distributed verbosely and symmetrically on his extremities, with the trunk, face, and neck spared (Figure 2A, Figure 2B, Figure 2C). Swelling in the right ulnar nerve and decreasing the sensation in the mentioned area was also observed. No neurological deficits were discovered.

**Diagnostic assessment:** bacilloscopy was done in slit skin smears and histological sections of the cutaneous hypochromic lesion to lead the investigation. It revealed granulomatous infiltrate with perineural and perivascular involvement, as well as a significant number of acid-fast bacilli. Together with the clinical presentation and laboratory results, these findings led to the diagnosis of lepromatous leprosy (Figure 2D).

**Treatment:** the patient was treated with Rifampicin, Clofazimine, and Dapsone, followed by 1 mg/kg/day prednisone for neuritis, and continued to improve his condition.

**Follow-up and outcome:** after these manifestations improved, the patient decided to come back to India. When he returned home, he was provided a confirmation letter of his diagnosis and strongly recommended seeking medical treatment and continuing treatment if needed.

## Discussion

Leprosy has been one of the world´s oldest diseases. Its prevalence has decreased globally since the first meeting of the World Health Organization´s Leprosy Expert Committee in 1956. Nonetheless, there are still endemic leprosy areas, such as India and Indonesia. In Saudi Arabia, while the precise number of infected patients is unknown, it does not pose a public health risk. Over 10-years from 2003 to 2012, Assiri and associations recorded 242 afflicted residents in Saudi Arabia [4]. These cases emphasize Saudi clinicians´ significance in keeping an eye out for nonendemic diseases like leprosy, considering the recent increase in immigration and ever-changing community composition.

Lepromatous leprosy may not display with the classic manifestations seen in most affected patients. In this regard, it should be mentioned that the first our case had a more visible clinical exam, with the existence of clinical manifestations typically associated with this type of disease with nose involvement. While the second patient had more slight symptoms with spots, and neural thickening could be the only symptoms noted during a clinical examination. For that, it was misdiagnosed on the first visit.

Leprosy is commonly misdiagnosed in low-prevalence areas because it can mimic other more popular skin disorders. Yang *et al*. reported a misdiagnosed case of leprosy that was initially treated as a tinea versicolor [5]. Similarly, our second patient was first misdiagnosed as *Tinea corporis*; nevertheless, he did not respond to treatment. This case shows the importance of maintaining a high index of suspicion for leprosy in the patients from endemic areas who present with lesions consistent with the disease.

The facial changes caused by lepromatous leprosy usually begin as chronic rhinitis. The manifestations are frequently so vague that leprosy is not diagnosed until the appearance of the skin lesions. Depth infections can cause septum perforation, and expanding to the surface of the hard palate can cause periostitis, which is noticeable on radiography. Clinically, more pathology has seemed when the nasal bones and the midline maxillary area are involved; erosion or destruction of the latter causes tissue collapse, with plunging and extending of the overlying skin and other surrounding tissues (saddle nose). This squall was seen in our first patient [6]. In 260 patients, Shehata *et al*. discovered the incidence rate of nasal involvement. They mentioned that the nose and septal perforation deformities appeared in 47% and 34% of patients, respectively [7].

In lepromatous leprosy, the neurologic involvements occur in an advanced stage. Additionally, it is spread slowly and symmetrical bilateral involvements with no relation to the skin manifestations [8]. In suspected cases, the first step is to prepare a slit-skin smear from a lesion to search for organisms. The histopathology of lepromatous leprosy is characterized by a priority of histiocytes with spindle-shaped form interlacing bands, whorls, or storiform patterns and a vast numeral of acid-fast bacilli. Overall, the epidermis layer covering the skin lesions is atrophied. According to the latest report, histopathological investigations could demonstrate the conventional lepromatous leprosy in the center of the lesions [9]. A skin biopsy of our patients has confirmed the diagnosis.

Investigating the contacts of leprosy patients is a significant policy since these permit earlier detection, inhibit the transmission chain, and avoid deformities and disabilities. Age between 5 and15, as well as age more than 30, had a high risk for diseases transmission. The actual transmission mechanism of leprosy is unknown. However, it was assumed to be spread through nasal discharge [10]. A thorough initial assessment is recommended in these cases, followed by careful checks [11], which were done in our patients. Regarding treatments, Immunosuppression with corticosteroids is the first-line treatment. Dapsone is another highly effective agent with limited side effects. The recommended regimen for leprosy is a combination of dapsone, rifampin, and clofazimine. Other treatment options such as ethionamide-prothionamide and streptomycin are limited due to liver toxicity of ethionamide-prothionamide and the need for parenteral administration of streptomycin [6,12].

**Patient perspective:** the patients were happy with the successful outcome of the treatment.

**Informed consent:** a written informed consent was obtained from the patients for participation in our study.

## Conclusion

Lepromatous leprosy can sometimes be challenging to diagnose, especially in low-prevalence areas. It is critical to prioritize the diagnosis on one's differential. Early detection and treatment of lepromatous leprosy are essential for minimizing the damage, restricting disability, and treating the disease proficiently.
